# Devastating Outcomes of Traditional Enemas: Unusual Indications for Well-Known Operations

**DOI:** 10.1055/s-0039-1700957

**Published:** 2020-02-08

**Authors:** Cleopatra Mshumpela, Giulia Brisighelli, Chris Westgarth-Taylor

**Affiliations:** 1 Department of Paediatric Surgery, University of the Witwatersrand Faculty of Health Sciences, Johannesburg, Parktown, South Africa; 2 Department of Paediatric Surgery, Chris Hani Baragwanath Hospital, Johannesburg, Gauteng, South Africa

**Keywords:** traditional enema, pull-through, PSARP

## Abstract

**Background** Despite serious health risks having been described, traditional enemas are still often used in African traditional medicine. We aim to report two cases of complications secondary to traditional enemas, to illustrate how severe the injuries can be, and to describe the use of a Swenson type endoanal pull-through and a posterior sagittal anorectoplasty (PSARP) as surgical options.

**Case Description** A 2-year-old girl presented with a necrotic rectum after a traditional enema administration. At admission, she required a laparotomy, colostomy fashioning, and extensive debridement of her rectum and perineum. She subsequently had a pull-through of the descending colon using a PSARP approach, a covering loop ileostomy, and a Malone Antegrade Continence Enema. The ileostomy was reversed at the age of 3 years of age and she is now clean with rectal washouts.

The second case was a one- and a half-year-old boy with full-thickness burns to the perineum and rectum secondary to a hot-water enema. A colostomy was initially brought out and pulled through 7 months post the initial surgery. He is now growing well and is fully continent to stools.

**Conclusions** The potential complications associated with the practice of administering at-home enemas can be quite devastating. A transanal pull-through and a PSARP have been proven to be successful techniques in patients who have suffered rectal burns due to traditional enemas.

## Introduction

African traditional medicine often includes the use of herbal or home-made enemas with a reported use of 89% in babies under 3 months of age in a study in Kwazulu-Natal. 1 The motivations for enema use vary in different cultures—from giving newborns enemas to “cleanse their meconium,” to treating constipation and administering herbal remedies that are too bitter to take orally. 1 At-home enema use is still very popular, even though serious health risks have been described, such as infections, dehydration, and injury to the rectal wall. 1 We present two cases that illustrate how extensive and potentially incapacitating these injuries can be. The aim of the study is to describe two procedures, derived from the posterior sagittal anorectoplasty (PSARP) for anorectal malformations and the pull-through for Hirschsprung's disease to address the long-term sequelae of traditional enema injuries.

## Case Report 1: Caustic Enema Injury

A 2-year-old girl presented with a 2-day history of perianal swelling and foul-smelling diarrhea post enema administration. The enema administration was initially denied and the ingredients of the herbal enema are unknown. Clinical examination revealed an acute abdomen and perianal discoloration with pus discharging from the anus. The white cell count was 9.89 × 10 ^9^ /L and the C reactive protein was 3; and the child had a normal renal function.

**Fig. 1:**
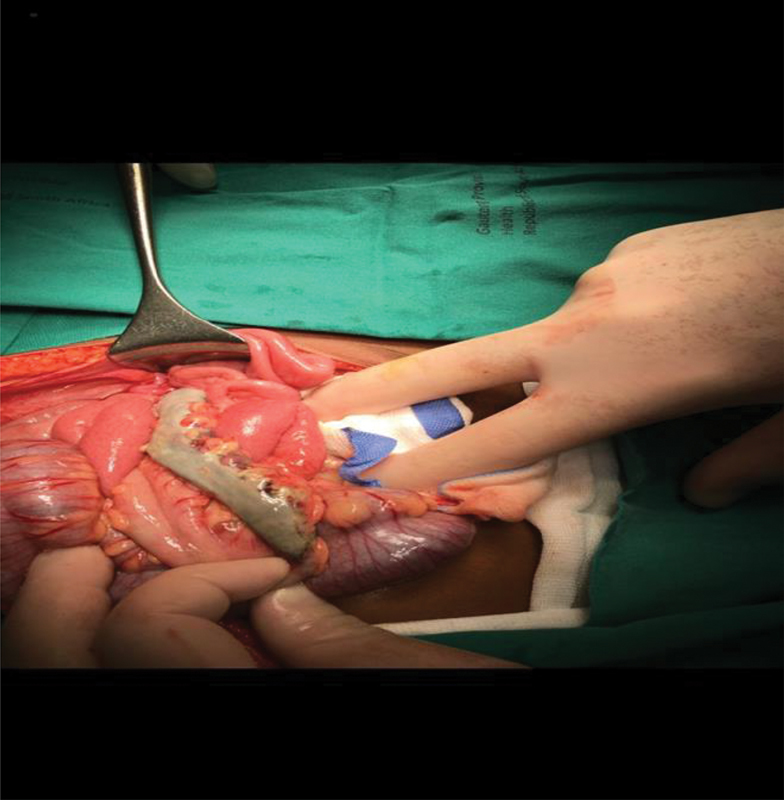
A 2-year-old during the first laparotomy post herbal enema administration—a necrotic rectum and sigmoid identified without any signs of perforation.

**Fig. 2:**
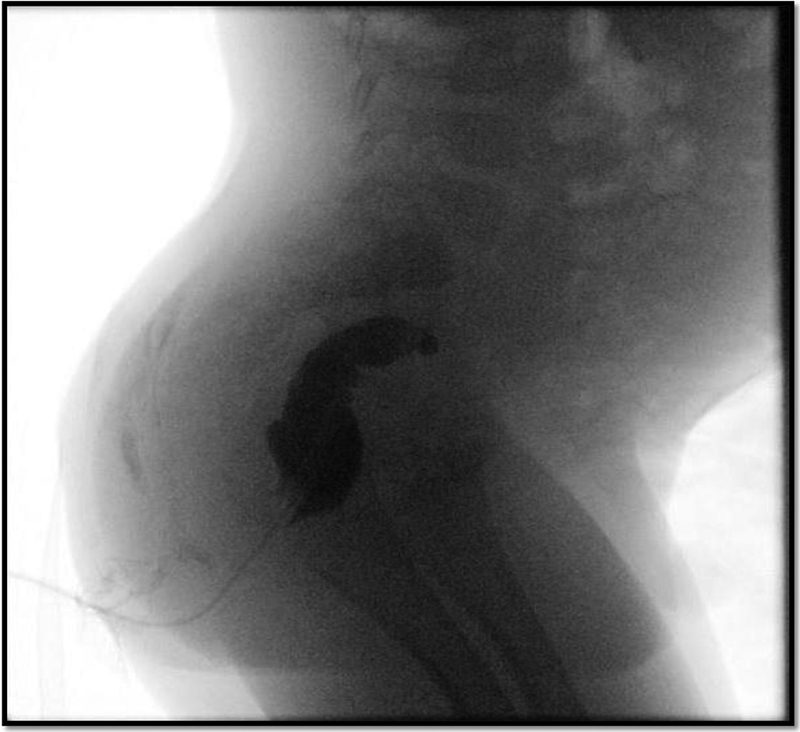
A 2-year-old at relook of the perineum for removal of packs 48 hours post laparotomy, resection of necrotic rectum, and sigmoid colon and end colostomy.

The child was taken to theater where examination of the perineum and anus revealed necrotic perianal skin and dentate line. At laparotomy, a necrotic rectum and sigmoid were identified without any signs of perforation ( Fig. 1 ). An end colostomy was brought out at the descending colon and the necrotic colon and rectum removed transanally. Packs were left in situ and the patient had a planned relook in 48 hours. At relook, further debridement of slough and necrotic muscle was performed ( Fig. 2 ). Histology showed transmural necrosis and serositis; no infection and ganglion cells were present throughout. Eight months post the diverting colostomy, the child had a completely closed anal opening with perineal scarring. The decision was made to perform a pull-through of the descending colostomy using a PSARP approach. A total body preparation was performed. Using a lower midline laparotomy, the stoma was taken down and the transverse colon was mobilized. Due to the extensive inflammatory reaction in the pelvic area, an iatrogenic posterior wall vaginal tear was caused during the dissection. The vaginal tear was located and repaired. After completing the pelvic dissection, the patient was positioned prone. Good muscular contractions were noted with stimulation (areas of sphincters marked) and a limited posterior sagittal incision was performed and deepened. The patient was subsequently moved back into supine position and the stoma end was pulled through the posterior sagittal incision. The anoplasty was performed after reconstruction of the muscle complex and perineal body. A Malone Antegrade Continence Enema (MACE) was fashioned in the right iliac fossa. A protective loop ileostomy was also brought out and reversed a month later, at the age of 3 years. The patient recovered uneventfully postoperatively and was discharged home to be followed up at the anorectal and stoma clinic. She was temporarily lost to follow-up and later represented with a complete stricture of the MACE. She was transitioned to rectal washout and is currently clean.

## Case Report 2: Hot-Water Enema Injury

**Fig. 3:**
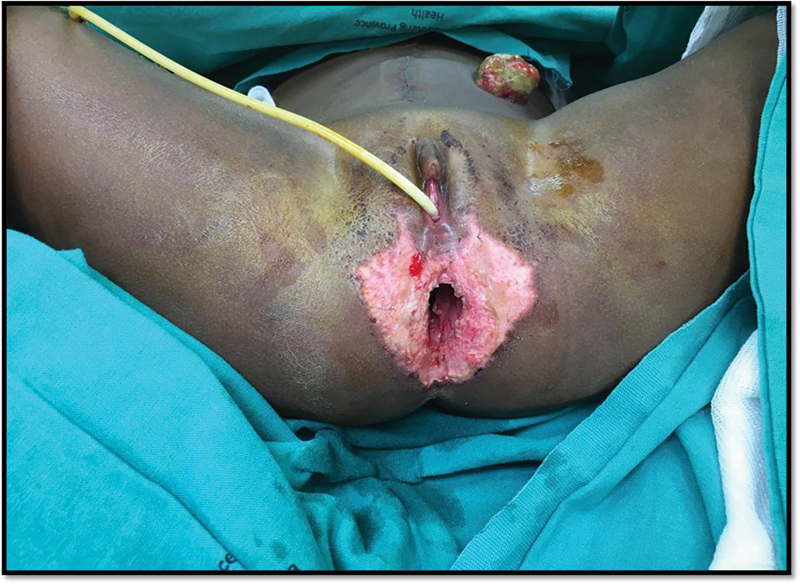
Contrast enema post hot-water enema administration showing a rectal stricture with total cutoff at the sacral promontory.

A 21-month-old male presented to another institution after a hot-water enema was administered for constipation. The child was fluid resuscitated and taken to theater the following day for an examination under anesthesia (EUA). He was found to have a 2 cm circumferential full-thickness burn, proximal to the dentate line, but involving the sphincter muscles and a left-sided abdominal mass was palpated. A laparotomy was therefore performed. No contamination in the peritoneal cavity was noted but there was severe inflammation of the rectum (up to the rectosigmoid junction) with areas of patchy necrosis. The mass palpated was found to be a pelvic kidney. An end colostomy was brought out. The patient was discharged 9 days post colostomy and referred to our center. At EUA, a rectal stricture was identified 4 cm proximal to the dentate line and a long colonic/rectal stricture was later confirmed on a contrast enema ( Fig. 3 ). The patient underwent a transanal pull-through 7 months after the diverting colostomy. A full body preparation was performed and the child was positioned prone in jack knife decubitus. A lone star was positioned and circumferential stay sutures were positioned 1 cm proximal to the dentate line. A Swenson-type dissection of the rectum and distal colon was performed transanally. The child was then positioned prone and the abdomen was entered through the old laparotomy wound. The Hartmann colostomy was taken down and the left colic vessels ligated (relying on the middle colic to provide blood supply) to achieve sufficient colonic length for the pull-through procedure. The distal bowel was mobilized and resected, the stoma was pulled through on the left, and a double layer coloanal anastomosis was fashioned. He recovered well after surgery and is now growing well and fully continent to stools.

## Discussion

In this report, we present two cases of unusual and devastating injuries caused by home enema administration. Both cases required emergency surgical intervention with local debridement and stool diversion. Ultimately, they underwent repair applying the principles used for the Swenson endoanal pull-through and the PSARP.

There are a few peculiar challenges that have been identified when operating on these cases. Acutely, a crucial step in the management of these children is fecal diversion. This needs to be performed as an emergency, to avoid peritoneal soiling secondary to possible perforation of the necrotic rectosigmoid and to avoid perineal necrotizing fasciitis. 2 3 The colostomy allows for the resolution of any sepsis before the definitive procedure can be performed. Following our experience with these two cases, in which severe or complete strictures of the anorectum have been observed, we advocate for a diverting colostomy, with a distal mucus fistula (if the bowel is not completely resected). A mucous fistula prevents a formation of a blind loop, should a stricture develop and it enables to later perform a distal colostogram to assess the condition and length of the distal bowel, if required.

Once the perineal and pelvic inflammatory reaction has settled, the patient might be amenable for definitive repair. Our patients had definitive repair at 7- and 8-month post diversion, but it is reasonable to think that 3 to 6 months post trauma could be an adequate time for the inflammation to resolve. A preoperative EUA could help assess the timing of the definitive repair.

We believe that counseling the parents extensively plays a crucial role before the reconstructive surgery. The families need to be aware of the risks of fecal incontinence to adjust their expectations and to be prepared for the need for daily washouts to keep the children mechanically clean. Regular follow-up is also very important after reconstructive surgery but it can be quite challenging in our setting mainly due to socioeconomic reasons such as lack of money for transport. Both our patients had poor prognostic factors for achieving fecal continence because of the involvement of their anal sphincters, the dentate line, and the extensive fibrosis in the area. Due to the significantly high risk of incontinence in the first case, compounded by absence of a rectum as a reservoir, a decision was made to fashion a MACE at the time of reconstruction.

The two techniques we used were derived from the Swenson type endoanal pull-through in the one case and a PSARP in the other case. Both these procedures are usually done in the neonatal period or in infancy. Operating on an older child has the disadvantage of a more rigid pelvis and therefore more difficult mobilization. 4 A thickened mesentery, inflamed mucosa, and a long-standing dilated colon have also been described as causes of some difficulties. 5 In our cases, the difficulty was further increased as we had to operate in a field with an enormous amount of scar tissue secondary to the extensive inflammatory reaction. This challenge has been reported by other authors when dealing with redo pull-through surgery. 6 Both procedures were successfully completed and both patients recovered uneventfully.

It is important to highlight the fact that these injuries are completely preventable and unacceptable. Public awareness with regard to the dangers and complications of at-home-enema use needs to be prioritized. This can be done tactfully, without disrespecting any cultural beliefs. Engaging and understanding the motivations for the continued use of these enemas could help in offering acceptable alternative solutions.

## Conclusion

The potential complications associated with the practice of administering at-home enemas are devastating. The first step would be to prevent these injuries by creating public awareness with regard to the potential dangers. When extensive rectal and perineal disruption is encountered, a diverting stoma needs to be performed. Fibrosis and severe scarring can be expected secondary to the severe inflammation. Although challenging, a full-thickness transanal pull-through and a PSARP have been proven to be successful techniques in patients who have suffered rectal burns due to traditional enemas. Fecal incontinence needs to be expected and addressed properly in these patients.

## Notes

New Insights & the Importance for the Pediatric Surgeon It is important to know the different surgical options that are available when children present with extensive injuries to the perineum and rectum.
